# Metabolomic Analysis Identifies Differences Between Wild and Domesticated Chili Pepper Fruits During Development (*Capsicum annuum* L.)

**DOI:** 10.3389/fpls.2022.893055

**Published:** 2022-06-13

**Authors:** Felipe Cervantes-Hernández, Neftalí Ochoa-Alejo, Octavio Martínez, José Juan Ordaz-Ortiz

**Affiliations:** ^1^Centro de Investigación y de Estudios Avanzados del Instituto Politécnico Nacional, Unidad de Genómica Avanzada, Irapuato, Mexico; ^2^Departamento de Ingeniería Genética, Centro de Investigación y de Estudios Avanzados del Instituto Politécnico Nacional, Unidad Irapuato, Irapuato, Mexico

**Keywords:** *Capsicum annuum*, global profiling, metabolomics, domestication process, fruit development

## Abstract

*Capsicum* spp. members are a rich source of specialized compounds due to their secondary metabolism. Some metabolic pathways have suffered modifications during the domestication process and improvement of agricultural traits. Here, we compared non-targeted LC–MS profiles from several areas: wild accessions (*C. annuum* L. var. *glabriusculum*), domesticated cultivars (*C. annuum* L.), and the F1 progeny of a domesticated, and a wild accession cross (in both directions) throughout seven stages of fruit development of chili pepper fruits. The main detected differences were in glycerophospholipid metabolism, flavone and flavonol biosynthesis, sphingolipid metabolism, and cutin biosynthesis. The domesticated group exhibited a higher abundance in 12′-apo-β-carotenal, among others capsorubin, and β-tocopherol. Palmitic acid and derivates, terpenoids, and quercitrin were prevalent in the wild accessions. F1 progeny showed a higher abundance of capsaicin, glycol stearate, and soyacerebroside I. This work supports evidence of the side-affectation of trait selection over the metabolism of chili pepper fruit development. Furthermore, it was also observed that there was a possible heterosis effect over the secondary metabolism in the F1 progeny.

## Introduction

During the plant domestication process, humans have selected and managed phenotypes of plants that presented desired characteristics for crop yield and agricultural conditions. This conscious or unconscious process, has resulted in changes in the breeding phenotypes and genotypes, making them more useful to humankind (Doebley et al., 2006; Clement et al., 2010; Luna-Ruiz et al., 2018).

*Capsicum annuum* is one of the most important horticultural crops worldwide (Qin et al., 2014; Guevara et al., 2021; Martínez-Ispizua et al., 2021). Belonging to the Solanaceae family, originated and putatively domesticated from *Capsicum annuum* L. var. *glabriusculum* in México (Pickersgill, 1997; Hernández-Verdugo et al., 2001; Martínez-Ispizua et al., 2021), this species is a rich source of phytochemicals and nutrients, such as flavonoids, carotenoids, and vitamins that are important for the human diet (Morales-Soto et al., 2013). In addition, the unique flavor and pungency are due to the existence of capsaicinoids, a group of alkaloids exclusively synthesized in the fruit of this plant species (Luna-Ruiz et al., 2018; Martínez-Ispizua et al., 2021).

The chili pepper domestication date is still under debate, but it has been estimated to be approximately 6,000 years BP based on the analysis of starch microfossils of domesticated accessions of chili pepper from seven Neotropical regions of America (Perry et al., 2007).

The domestication process resulted in a wide diversity of morphological, and phytochemical traits among the genus *Capsicum* (Hernández-Verdugo et al., 2001; Pickersgill, 2007; Qin et al., 2014). Some of the most appreciable changes in cultivars are the color, size, and form of the fruits (Hernández-Verdugo et al., 2001; Pickersgill, 2007; Qin et al., 2014; Solomon et al., 2021). Specialized metabolites from secondary metabolisms, such as capsaicinoids, carotenoids, and flavonoids, pass through alterations during domestication (Luna-Ruiz et al., 2018). All the latter are extensively reported to present shifts in their abundance across cultivars (Paran and van der Knaap, 2007; Wahyuni et al., 2011; Qin et al., 2014).

Wild accessions conserve high levels of genetic variation (Hernández-Verdugo et al., 2001; Hayano-Kanashiro et al., 2016) and thus it is reflected in their ability to adapt to different environments or diseases. Hernández-Verdugo et al. (2001), reported a wild accession (*C. annuum* var. *glabriusculum*) from the Norwest of México that was resistant to the *Pepper huasteco virus* (PHV). In contrast, most of the domesticated cultivars are poorly adapted to stressful environments; however, they are resistant to parasites and diseases. The domesticated cultivar “Criollo de Morelos landrace CM334” is a successful case of a genetic resistant inbred to *Phytophthora capsici* and nematodes (Pegard et al., 2005; Luna-Ruiz et al., 2018).

To date, shifts in phytochemical profiles of *C. annuum* have been minorly explored from a holistic perspective. Most of the metabolites in *C. annuum* remain unknown (Luna-Ruiz et al., 2018). Some metabolites have been reported for *C. annuum* L. species, for example, Pascale et al. (2020), outlined sixteen quercetin glycoconjugates in peperoni di Senise peppers; among them, five which were tentatively identified as quercetin-(galloyl-rhamnoside)-hexoside, quercetin-(sinapoyl-hexoside)-rhamnoside, quercetin- (galloyl-caffeoyl-hexoside)-rhamnoside, quercetin-(feruloyl-hexoside)-rhamnoside, and quercetin-(succinyl-rhamnoside)-rhamnoside, were reported for the first time. In another study, Morales-Soto et al. (2013) reported a total of 45 compounds tentatively identified mainly as glycoside derivatives of flavonoids such as quercetin 3,7-di-*O*-α-L-rhamnopyranoside and narigenin-7-*O*-β-D-(3″-*p*-coumaroyl)-glucopyranoside. Likewise, other polar metabolites such as organic acids, nucleosides, and amino acids were also detected. More recently, Guevara et al. (2021), reported the identification of compounds with potential therapeutic uses in fruits from sweet pepper (*C. annuum* L.) and their modulation by nitric oxide; 12 differential bioactive compounds were identified including quercetin and its derivatives, L-tryptophan, phytosphingosin, FAD, gingerglycolipid A, tetradydropentoxylin, blumenol C glucoside, colnelenic acid and capsoside A.

Fratianni et al. (2020), measured the amounts of bioactive compounds from different Campania native sweet pepper varieties. Polyphenols (caffeic, ferulic, *p*-coumaric, gallic, and chlorogenic acids, catechin, epicatechin, quercetin, rutin, naringenin, and apigenin) ranged between 1.37 nmol g^–1^ and 3.42 mmol g^–1^; β-carotene was abundant in a red variety, while yellow and red varieties showed a content of ascorbic acid not inferior to 0.82 mg ^g–1^, and in some varieties, the content of ascorbic acid was almost inconsistent. In the same manner, Ribes-Moya et al. (2020) reported the variation in flavonoids in a collection of chili peppers (*Capsicum* sp.) under organic and conventional cultivation practices. Their main results explained that luteolin and quercetin showed the highest contribution to the total phenolic content at both, unripe and fully ripe states evaluated, while myricetin, apigenin, and kaempferol showed lower contributions.

This study focused on exploring the metabolite diversity, and differences between domesticated and wild accessions of *Capsicum annuum* L. during fruit development. Furthermore, an F1 progeny between a domesticated and a wild accession was considered. This investigation showed a differential clustering and presence of metabolites related to terpenoid and fatty acid classifications in domesticated and wild accessions of chili pepper suggesting a possible impact of domestication.

## Materials and Methods

### Chemicals and Reagents

All the chemicals and reagents were purchased from AccesoLab (AccesoLab S.A. de C.V., Mexico, Mexico). Formic acid, methanol, and acetonitrile were MS grade and purchased from Sigma-Aldrich (Merck S.A. de. CV., Mexico, Mexico).

### Plant Material and Fruit Harvesting

Seeds from each chili pepper accession (*C. annuum* L.; Table 1) were treated with 70% ethanol solution before treatment with 10% hypochlorite solution for 10 s, followed by six rinses with deionized water. Wild accession seeds were treated first with 50% of the sulfuric acid solution for scarification. All accession seeds were germinated using a mixture of peat moss, perlite, sludge, forest soil, and vermiculite in a plastic tray and incubated in a chamber maintained at 23–25°C. Three-week-old plants were individually transplanted to 5 L pots at greenhouse facilities in optimum conditions (30–32°C) and fertilized with Long Ashton solution every two weeks. Flowers were labeled and fruits from 10 different plants of each accession were collected from 0 to 60 days after anthesis (DAA) at intervals of 10 days during development. At least, 10 fruits and flowers were collected from each plant. All the collected fruits were immediately frozen in liquid nitrogen and stored at −80°C.

**TABLE 1:** List of chili pepper (*C*. *annuum*) accessions used.

Accession	Group	Key
*C. annuum* L. cv. Tampiqueño 74	Domesticated	ST
*C. annuum* L. cv. Criollo de Morelos 334	Domesticated	CM
*C. annuum* L. cv. California Wonder	Domesticated	CW
*C. annuum* L. cv. Zunla-1	Domesticated	ZU
*C. annuum* L. var. *glabriusculum* (Querétaro)	Wild	QU
*C. annuum* L. var. *glabriusculum* (Coahuila)	Wild	CO
F1 *C. annuum* L. CM♂ X QU♀	Cross	F1-CQ
F1 *C. annuum* L. QU ♂ X CM ♀	Cross	F1-QC

### Metabolite Extraction

The flowers and whole fruits were grounded manually in a mortar with a pestle using liquid nitrogen. Following this process, two biological replicates were made for each developmental stage. A biological replicate comprised of at least two fruits harvested from 2 to 6 individual plants, depending upon the developmental stage and cultivar.

For the metabolite extraction, a modified Matyash method was employed as outlined previously (Matyash et al., 2008; Cervantes-Hernández et al., 2019): 1.5 ml of methanol was added to 100 mg of frozen sample in a test tube. After 1 min vortexed, 5 ml of diethyl ether were added. Samples were incubated at room temperature with gentle stirring for 1 h and then 1.5 ml of ultra-pure water (18 Ω, milli-Q system) were added and vigorously mixed for 1 min. The mixture was settled at room temperature for 10 min, and then centrifuged at 1,000 × *g* for 10 min. The organic phase was collected for this study. Samples were vacuum dried (miVac, Genevac) at 30°C for 30 min and stored at −80°C until further analysis.

### LC–MS Analysis

In total, five artificial quality control (QC) samples were prepared to verify the instrument drift and calibration along with the analysis in UHPLC-QTOF-HRMS. Each QC sample contained an equative portion of all the extracted samples in the experiment.

For UHPLC–MS analysis, all the samples were resuspended in 2 ml of acetonitrile/ultra-pure water 50:50 (v/v) and filtered using individual pre-packed filters (0.2 μm, PTFE, Agilent Technologies, Santa Clara, United States). Samples were injected by a randomized list order on a UPLC^®^ (Acquity class I, Waters, Milford, CA, United States) coupled with an orthogonal QTOF mass spectrometer (SYNAPT G1 HDMS, Waters, Milford, CA, United States). Chromatographic separation was achieved on a reversed-phase CSH C18 column (2.1 mm × 150 nm, 1.7 μm, Waters, Milford, CA, United States) at 35°C during chromatographic separation. Mobile phase A was composed by ultra-pure water with formic acid (0.1%; v/v); Mobile phase B was acetonitrile with formic acid (0.1%, v/v) with a flow rate of 0.3 ml/min following a gradient method: from 0 to 0.5 min, 35% B; 0.5 to 10 min, 35–65% B; 10 to 30 min, 65–98% B; 30 to 31 min, 98% B; 31 to 31.5 min, 98–99% B; 31.5 to 33 min, 99% B; 33 to 33.1 min, 99–35% B; 33.1 to 36 min, 35% B. The mass spectrometer range was set from 50 to 1,500 Da. Both ionization modes were injected separately. For positive electrospray ionization (ESI) mode, the conditions were set as follows: capillary voltage 3 kV; cone voltage 40 V; source temperature 120°C; desolvation temperature 350°C; desolvation gas flow 500 L/h. For the negative ESI mode: capillary voltage 2.5 kV; cone voltage 40 V; source temperature 120°C; desolvation temperature 300°C; desolvation gas flow 500 L/h. Leucine–enkephalin (2 ng/ml) was infused as LockSpray reference internal mass calibrant at a flow rate of 5 μl/min and its signal was monitored every 10 s. Data were collected in a continuum mode with an MS scan time of 1.5 s. In both ionization modes, data were acquired in MS^e^ experiments using Ar as the collision gas with collision energy in the trap region of 10 eV (Function 1, low energy) and ranging from 20–50 eV (Function 2, high voltage).

### Data Analysis

Positive and negative electrospray ionization data were independently analyzed using Progenesis QI for small molecules software (Non-Linear Dynamics, Waters, Milford, MA, United States). Alignment, normalization, and deconvolution were set at standard parameters. Pre-identification was performed using Chemspider Databases (PlantCyc, Plant Metabolic Network, KEGG, HMDB, and ChEBI) and with an in-house database with a minimum match of 80% for precursor ions, MS/MS data, retention times values, and isotope distribution were included for increasing match score values. Statistics and graphics were performed using R software (3.3.3v, Vienna, Austria). A *t*-test was used for significance analysis (*p*-value < 0.05). Clustering analysis was performed using the “pvclust” package in R, using Euclidean distance and Ward’s linkage method for clustering. Values of bootstrap probability (bp) and approximately unbiased *p*-value (au) are parameters related to the grouping quality (Suzuki and Shimodaira, 2006). Compounds were classified according to their compound classes using the ClassyFire tool (Djoumbou Feunang et al., 2016). MetaboAnalyst (Pang et al., 2021) was used for principal component analysis, pathway analysis, and heatmap visualization. The data for principal component analysis (PCA) was first scaled to the Pareto method and submitted subsequently to PCA. The biosynthetic pathway analysis was performed using the *Arabidopsis thaliana* database of KEGG and pre-annotated compound names were used for the query. A heatmap was elaborated using normalized values of all the pre-annotated compounds during the development. A complete heatmap of the dataset is shown in Supplementary Figure 1. OPLS–DA and S-plots were generated with scaling data to Pareto using Ezinfo 3.0.3.0 software (Umetrics).

## Results

### Metabolite Pre-identifications During Fruit Development of *C. annuum*

In total, 122 samples of pepper fruit extract from different accessions at different developmental stages were analyzed in both the positive and negative ionization modes. Chromatographic data were processed, aligned, and normalized using Progenesis QI for small molecules. A total of 456 and 292 features or m/z values were detected in positive and negative modes, respectively. Supplementary Figure 2 shows some examples of pre-identified metabolite distribution in QC samples per ionization mode.

According to the current levels of confidence for metabolite annotation (Blaženović et al., 2018; Reisdorph et al., 2019), around 160 putative compounds level 2 were annotated with a match score of over 80% which considered the exact mass of precursor ions m/z, precursor ion isotopic pattern, and fragmentation spectra matching. A complete dataset of putative compounds and unknown features is presented in Supplementary Table 1, containing metabolite ID, description, compound class, acquisition mode, and averaged abundance per developmental stage.

### Pathways Analysis Highlights Glycerophospholipids Metabolism During the Development of Chili Pepper Fruits

**FIGURE 1:**
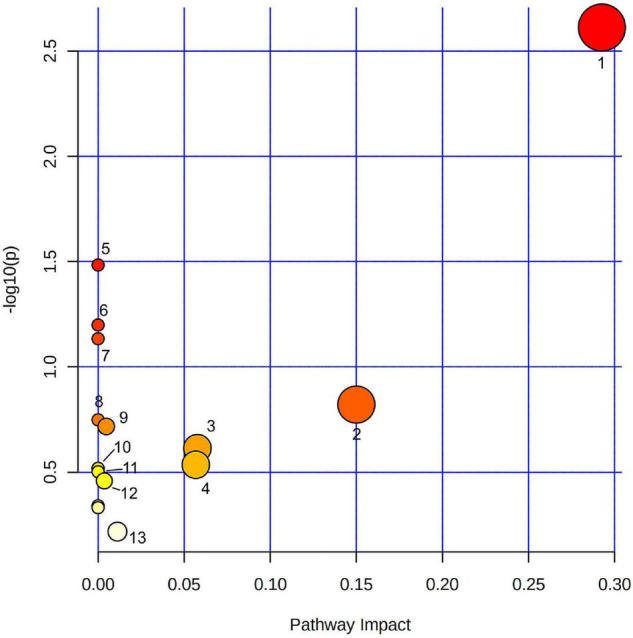
Pathway analysis of pre-annotated compounds in fruits at different developmental stages from domesticated and wild accessions of chili pepper using LC–MS. 1. Glycerophospholipid metabolism; 2. Flavone and flavonol biosynthesis; 3. Sphingolipid metabolism; 4. Glycerolipid metabolism; 5. Cutin, suberin, and wax biosynthesis; 6. Linoleic acid metabolism; 7. α-Linoleic acid metabolism; 8. Arachidonic acid metabolism; 9. Glycosylphosphatidylinositol (GPI)-anchor biosynthesis; 10. Biosynthesis of unsaturated fatty acid; 11. Fatty acid elongation; 12. Phosphatidylinositol signaling system; and 13. Fatty acid biosynthesis. Color represents –log10(*p*) value, size correlates with pathway impact value.

Using all 158 pre-annotated compound identifications (Figure 1), a biosynthesis pathway analysis was performed. In total, 19 pathway classifications were found in the dataset, where glycerophospholipids metabolism was significantly represented among the other categories according to the impact and *p*-value (Figure 1). Compounds in this category were PC [18:3(9Z,12Z,15Z)/18:3(6Z,9Z,12Z)], PE [18:2 (9Z, 12Z)/16:0]; LysoPC (15:0), and LysoPA (0:0/16:0). Other metabolic pathways, such as flavone and flavonol biosynthesis, linoleic acid metabolism, α-linoleic acid metabolism, sphingolipid metabolism, glycerolipids metabolism, and cutin, suberin, and wax biosynthesis were also highlighted based on the impact value, but they did not pass the standard significant threshold (*p*-value < 0.05). Arachidonic acid metabolism, glycosylphosphatidylinositol (GPI)-anchor biosynthesis, glycerolipid metabolism, biosynthesis of unsaturated fatty acids, fatty acid elongation, phosphatidylinositol signaling system, fatty acid degradation, ubiquinone, and another terpenoid–quinone biosynthesis, phenylpropanoid biosynthesis, porphyrin, and chlorophyll metabolism, fatty acid biosynthesis showed only one compound per category, that resulted in a low impact and non-significant *p*-value for the analysis.

### Clustering Analysis Segregates Chili Pepper Fruits Depending on Their Domestication Category

**FIGURE 2:**
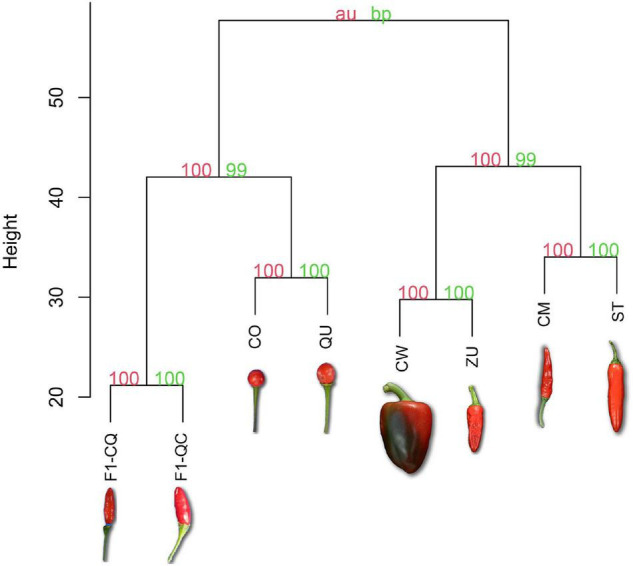
Hierarchical clustering of mean values from 748 features during development of wild, domesticated, and cross F1 accession of *C. annuum*. Bootstrap probability (bp) appears in green; approximately unbiased *p*-value (au) appears in red. The orientation of fruits in the photo represents if they are erect or pendent in the plant. The distance was measured using Euclidean and Ward’s linkage method for clustering.

Bootstrapped hierarchical analysis of metabolic features during the whole development of chili pepper fruits showed clustering of the different accessions based on their domestication level. Figure 2 shows the values of bootstrap probability (green) and approximately unbiased *p*-values (red) in each bifurcation. Domesticated cultivars clustered exclusively with other domesticated cultivars. The other big clade was constituted by the wild accessions and the F1 progenies from crosses, but even these exhibited a separation based on their similarities. F1-QC and F1-CQ were the most distant groups from the full dataset of the experiment.

**FIGURE 3:**
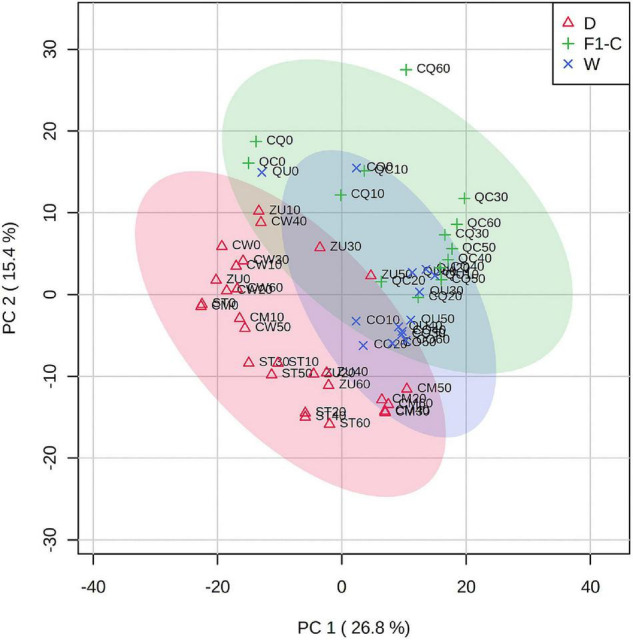
Principal component analysis of fruits at different developmental stages from domesticated or wild accessions of *C. annuum*. Component 1 = 26.8%; component 2 = 15.4%. Color meaning is indicated in the legend. The developmental stage is indicated as two digits after the accession key, for example, ST0 for Tampiqueño74 stage 0 (flowers); ST20 Tampiqueño74 at 20 DAA.

Predominantly based on the domestication level, a 2D principal component analysis (Figure 3) shows consistent clustering of domestication level along the developmental stages, which is also coherent with the clustering analysis presented in Figure 2. Only metabolomic profiles from 00 DAA were more dispersed from other stages of development, suggesting that flower metabolites were different compared with the fruit for each accession. A total of 42.2% of the variance was explained using the two components.

### Significant Metabolite Differences During Fruit Development of Wild and Domesticated *C. annuum*

**FIGURE 4:**
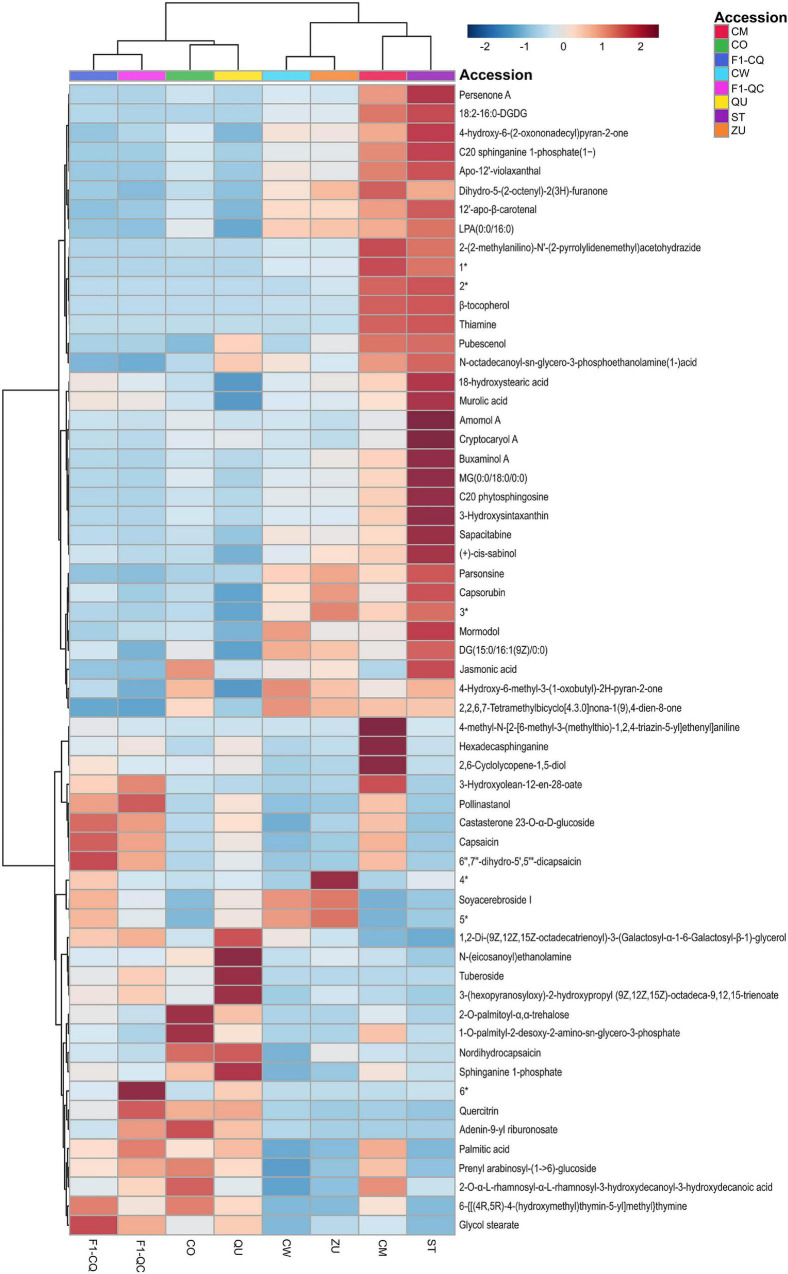
Heatmap of pre-annotated compounds in *Capsicum annuum* during fruit development. 1* Glycerol 2-(9Z,12Z-octadecadienoate)1-hexadecanoate3- *O*-[α-D-galactopyranosyl-(1- > 6)-β-D-galactopyranoside]; 2* 2,5-epoxy-2β-hydroxy-8α-(2-methylbut-2-enoyloxy)-4(15),10(14),11(13)-germacratrien-12,6α-olide; 3* N-(4-{[(2-amino-4-oxo-1,4,5,6,7,8-hexahydro-6-pteridinyl)methyl]amino}benzoyl)-γ-glutamyl-γ-glutamylglutamic acid; 4* 3-{[(2,3-dihydroxypropoxy) (hydroxy)phosphoryl]oxy}-2-(palmitoyloxy) propyl (9E,12E)-9,12-octadecadienoate; 5* (2S)-3-(β-D-galactopyranosyloxy)-2-[(9Z,12Z)-9,12-octadecadienoyloxy]propyl (9Z,12Z,15Z)-9,12,15-octadecatrienoate; 6* 2,6,6-trimethyl-4-[[3,4,5-trihydroxy-6-(hydroxymethyl)-2-oxanyl]oxy]-1-cyclohexenecarboxaldehyde. Euclidean distance and Ward’s linkage method was used for clustering.

Figure 4 displays the heatmap of the 60 top discriminant pre-annotated compounds with abundance scaled and grouped by accession. Dendrograms at the top and left side of Figure 4 clustered the accessions and features, respectively. The accession dendrogram was consistent with the whole dataset dendrogram. It presented two big clades: one was exclusive for domesticated accessions and the other clade was constituted by the wild and F1 cross accessions.

The upper clade of the pre-annotated compound dendrogram represents the more representative abundance in domesticated *C. annuum* fruits. It was composed of jasmonic acid, capsorubin, 12′-apo-β-carotenal, β-tocopherol, murolic acid, LPA (0:0/16:0), 4-hydroxy-6-methyl-3-(1-oxobutyl)-2H-pyran-2-one, dihydro-5-(2-octenyl)-2(3H)-furanone, parsonsine, C20 sphinganine 1-phosphate, C20 phytosphingosine, and thiamine, among others.

Capsaicin and some other capsaicinoids were more abundant in wild and F1 cross accessions (bottom clade). In addition, we found sphinganine, quercitrin, castasterone 23-*O*-α-D-glucoside, palmitic acid and derivates, N-(icosanoyl) ethanolamine, tuberoside, sphinganine 1-phosphate, prenyl arabinosyl-(1- > 6)-glucoside, soyacerebroside I, glycol stearate, and hexadecasphinganine as richer compounds in these groups.

In the F1 progenies cluster, (18R)-18-hydroxynonadecanoic acid, gypsogenin 3-*O*-β-D-glucuronide, pollinastanol, theasapogenol E were more abundant in contrast with the other two groups, domesticated and wild accession.

**FIGURE 5:**
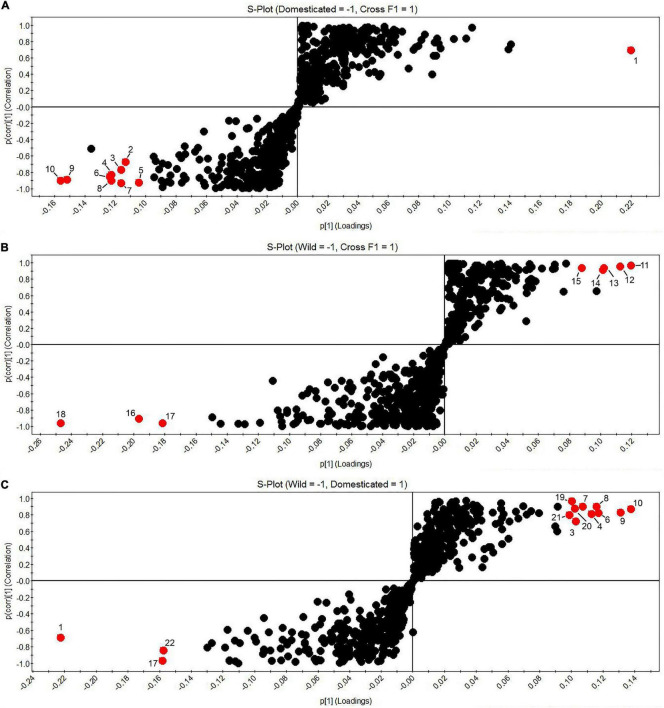
S-plots. **(A)** Domesticated (left) vs. F1 progenies (right); **(B)** Wild (left) vs. F1 progenies (right); **(C)** Wild (left) vs. Domesticated (right). 1. UNK19.30_269.2689n; 2. UNK27.39_311.1710m/z; 3. 4-hydroxy-6-(2-oxononadecyl)pyran-2-one; 4. UNK26.15_337.3352n; 5. UNK24.68_309.3061n; 6. UNK26.98_395.3129m/z; 7. UNK23.37_283.2895n; 8. UNK27.29_363.3516n; 9. 12′-apo-β-carotenal, 10. dihydro-5-(2-octenyl)-2(3H)-furanone; 11. UNK33.34_638.4883n; 12. UNK23.32_414.3364n; 13. Capsaicin; 14. UNK2.77_583.2669n, 15. UNK2.90_612.2727m/z; 16. UNK33.96_859.6947n; 17. UNK1.41_678.5108n; 18. UNK30.56_774.5371n; 19. UNK27.83_311.3200n; 20. UNK19.55_405.2257m/z; 21. UNK15.76_299.2018m/z; and 22. UNK7.59_137.0603m/z.

S-plots (Figure 5) were used to pair-comparison between the different accessions based on their domestication category. This plot is a powerful visualization method for comparing two conditions. S-plot considered, for the *y*-axis, the values of the correlation coefficient of the first component and the concentration of any feature; the *x*-axis is referred to the covariance of the first principal component and concentration of each feature (Pan et al., 2021). The red dots in Figure 5 represent the significant features (*p*-value < 0.05 and correlation > 60%) in each group. 12′-apo-β-carotenal, and 4-hydroxy-6-(2-oxononadecyl) pyran-2-one were significantly pre-annotated compounds in the domesticated group. Besides, the wild group presented significance only in features unfortunately no pre-identification. F1 cross accessions exhibited capsaicin as a significant compound compared with the domesticated cultivars but not against the wild group.

## Discussion

Metabolic global profiles of chili pepper fruit (*C. annuum*) have been reported previously by different authors (Wahyuni et al., 2011, 2013b; Fratianni et al., 2020), including tissue-specific (Cervantes-Hernández et al., 2019) and fruits at different developmental stages (Jang et al., 2015). However, to the best of our knowledge, this is the first study that aimed at exploring comparatively the metabolomic profiles during fruit development in domesticated and wild accessions of chili pepper fruits.

Hierarchical cluster analysis (Figure 2) of features averaged intensities across the seven developmental stages showed clustering of the accessions based on their domestication level. The high values of bp and au demonstrated high reliability of the grouping clustering (Suzuki and Shimodaira, 2006). Domesticated accessions formed a big clade, suggesting alterations in the phytochemical profiles probably because of the domestication process and selection of different traits. The other main clade was outlined by the wild and F1 cross-group, which phenotypically was more similar than that of the domesticated accessions. Also, F1-QC and F1-CQ showed segregation from the two wild accessions in the superior clade.

The separation of the first filial progeny could suggest a potential heterosis event. Compounds related to the secondary metabolism, specifically capsaicinoids, were found more abundant in F1 crosses. This shift in plant defense mechanism could represent an advantage over the progenitors in the wild. Also, gypsogenin 3-*O*-β-D-glucuronide and theasapogenol E, are two saponins that were more abundant in the F1 group. It is also known that saponin compounds have a role in plant defense. Other authors have demonstrated the behavior of the genetic heritability in the F1 progeny using different plant species. Superior traits such as fruit yield per plant, fruit number, and fewer days for fruit ripening have been observed in the F1 progeny between wild and domesticated accessions (Geleta et al., 2004; Marame et al., 2009; Rodrigues et al., 2012).

Our results, using a biosynthetic pathway analysis, demonstrated a richer abundance of lipids and hydrophobic compounds in all the analyzed samples. Lipids in plants, as in other organisms, are essential for cell membrane generation, a source of energy, and act as signal molecules (Janda et al., 2013; Aid, 2019). Lipids also have a role as a precursor of important metabolic pathways. Fatty acids are precursors in the biosynthesis of secondary metabolites, such as isoprenoids and capsaicinoids (Arce-Rodríguez and Ochoa-Alejo, 2019; Schmid, 2021). Glycerophospholipids metabolism was the category with the highest impact and lower *p*-value among the most representative metabolic pathways. Our domesticated group exhibited a significant abundance of these compound classes compared with the wild and cross F1 groups. This could probably be related to the modifications in the cell membrane structure of fruit pericarp. In *Capsicum annuum* L. cv. Bell pepper, Sutliff et al. (2021) reported the presence of 57 glycerophospholipids and glycerolipids compounds in the mature fruits. The selection of fruit color traits could be associated with the modification of nutritional characteristics in bell pepper (Sutliff et al., 2021). Some authors reported that this compound group is involved in different biological processes, such as plant defense, adaptation, and fruit water loss resistance, and probably resulted as an effect of the domestication process (Parsons et al., 2013; Villa-Ruano et al., 2018).

Previous studies have shown that fatty acids were possibly affected as a selected trait during the domestication process. Cuticle lipid composition seems to be unknowingly modified by this artificial selection by breeders with the intention of improving the appearance of the fruit and reducing fruit water loss (Parsons et al., 2013). Dormancy of fruit seeds is a trait of interest for producers, and they may have altered the profile of fatty acids. It has been reported that seeds of the domesticated chili pepper do not exhibit dormancy (Luna-Ruiz et al., 2018). Besides, wild chili pepper seeds have thick lignified test as to preserve water during drying (Luna-Ruiz et al., 2018). In addition, in different species of legumes, it seems that the domestication process has led to a decrease in the content of linoleic acid, another common lipid in plant tissues (Fernández-Marín et al., 2014). Guevara et al. (2021) reported the presence of higher levels of phytosphingosine, mainly in red/mature, than those in the green chili pepper fruit, and their implications in the ripening process.

Flavone and flavonol compounds belong to flavonoids, a large group of molecules in plants (Howard et al., 2000; Wahyuni et al., 2013a). Flavones present a 2-phenylchromen-4-one backbone and, flavonols bear a hydroxy group at the 3-position (Wahyuni et al., 2013a). Here, we pre-annotated some of these compound classes: such as quercitrin, neoflavan, and Kaempferol 3-xylosylglucoside. Different authors have reported this pathway as one of the most affected during the domestication and selection of chili pepper (Luna-Ruiz et al., 2018). It has been reported that almost all the flavonoid levels vary among cultivars and the fruit maturation stage (Morales-Soto et al., 2013). Wahyuni et al. (2014) reported an epistatic effect on semi-polar metabolites, including flavonol *O*-glycosides, in F2 segregation of crossing *C. annuum* AC1979 (female) and *C. chinense* (male) (Wahyuni et al., 2014).

There are several published reports that focus on pungent compounds and their related genes in chili pepper fruit. Without hesitation, this is one of the most attractive properties of the fruit. Therefore, the domestication process has had a relevant repercussion on the selection of traits that increase or decrease the content of capsaicinoids (Luna-Ruiz et al., 2018). In the wild, these metabolites function as a repellent against seed predators, such as small mammalians.

In the S-plots (Figure 5), 12′-apo-β-carotenal, a hydrophobic terpene of the group of apocarotenoids, is shown to be significant in the domesticated group. Zoccali et al. (2021), published the profiles of apocarotenoids in different accessions of *Capsicum.* They identified 19 free apocarotenoids and 8 apocarotenoids fatty acid esters, including 12′-apo-β-carotenal. Terpenoids in plants have different defensive functions as cytotoxic, phototoxic, and phytoalexins against microbials and insects (Cheng et al., 2007; Bakkali et al., 2008; Pandey et al., 2017). They are also attributed to attracting pollinators and seed dispersers (Langenheim, 1994). However, little is known about the terpenoids involvement in affectation during the domestication process in fruits.

Different authors agree that the domestication process has driven a decrease in metabolic diversity, mainly related to adaptation and pathogen resistance. Meyer et al. (2012), published a review about more than 200 different crops where wild progenitors showed a bigger abundancy in specialized metabolites, mainly related to flavor, pigments, and toxicity (Meyer et al., 2012).

The domestication of *C. annuum* plants changed the form, size, pungency, and color of the fruit based on the specific desired traits. Unfortunately, this artificial selection has also caused modifications in non-specific metabolism. While our results showed an increase in the abundance of lipid metabolism in domesticated cultivars, they also demonstrated a decrease of capsaicinoids, terpenoids, flavones, and flavonol compounds when compared with their wild ancestors. The resulting F1 progenies between the wild (QU) and the domesticated (CM) chili peppers (crosses in both directions) displayed a possible case of heterosis in genes related to the defensive strategies in the plants. It is our view that increasing the metabolomic analysis in a bigger number of accessions and cultivars will improve the resolution of the present results. Furthermore, the generation and comparison of additional metabolomic data, combined with molecular data, during the fruit development, could increase our understanding of the effects that occur due to the domestication process. This would enable the development of new genetic strategies to bring back metabolic diversity.

## Data Availability Statement

The original contributions presented in this study are included in the article/Supplementary Material, further inquiries can be directed to the corresponding author/s.

## Author Contributions

FC-H performed the experiments and wrote the manuscript. JO-O and OM designed and supervised the experiment. FC-H and OM analyzed the data. FC-H, JO-O, OM, and NO-A interpreted the results. JO-O, OM, and NO-A revised and edited the manuscript. All authors contributed to the article and approved the submitted version.

## Conflict of Interest

The authors declare that the research was conducted in the absence of any commercial or financial relationships that could be construed as a potential conflict of interest.

## Publisher’s Note

All claims expressed in this article are solely those of the authors and do not necessarily represent those of their affiliated organizations, or those of the publisher, the editors and the reviewers. Any product that may be evaluated in this article, or claim that may be made by its manufacturer, is not guaranteed or endorsed by the publisher.
