# Supplementary material for: Metabolomic Analysis Identifies Differences Between Wild and Domesticated Chili Pepper Fruits During Development (Capsicum annuum L.)
Source: Front Plant Sci. 2022 Jun 13;13:893055. doi: 10.3389/fpls.2022.893055 (PMC9234519; doi:10.3389/fpls.2022.893055)
Supplement: Supplementary file 1 [file Data_Sheet_1.PDF]

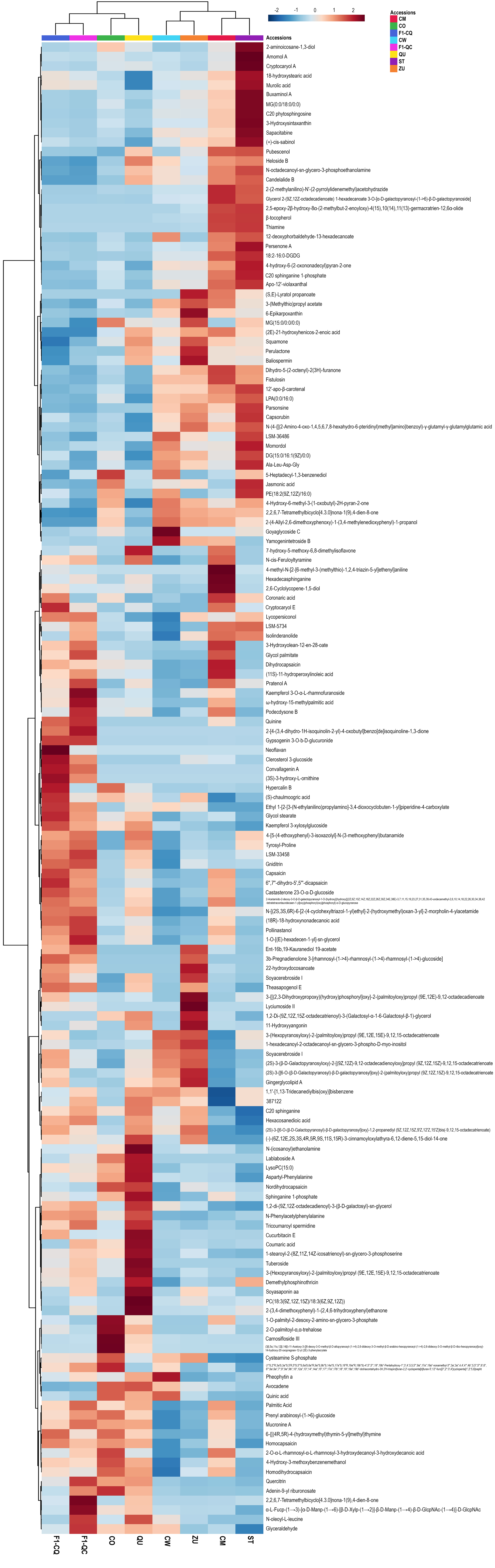

-2
-1
0
1
2
Accessions
CM
CO
F1-CQ
CW
F1-QC
QU
ST
ZU
Accessions
2-aminoicosane-1,3-diol
Amomol A
Cryptocaryol A
18-hydroxystearic acid
Murolic acid
Buxaminol A
MG(0:0/18:0/0:0)
C20 phytosphingosine
3-Hydroxysintaxanthin
Sapacitabine
(+)-cis-sabinol
Pubescenol
Heloside B
N-octadecanoyl-sn-glycero-3-phosphoethanolamine
Candelalide B
2-(2-methylanilino)-N'-(2-pyrrolylidenemethyl)acetohydrazide
Glycerol 2-(9Z,12Z-octadecadienoate) 1-hexadecanoate 3-O-[α-D-galactopyranosyl-(1->6)-β-D-galactopyranoside]
2,5-epoxy-2β-hydroxy-8α-(2-methylbut-2-enoyloxy)-4(15),10(14),11(13)-germacratrien-12,6α-olide
β-tocopherol
Thiamine
12-deoxyphorbaldehyde-13-hexadecanoate
Persenone A
18:2-16:0-DGDG
4-hydroxy-6-(2-oxononadecyl)pyran-2-one
C20 sphinganine 1-phosphate
Apo-12'-violaxanthal
(S,E)-Lyratol propanoate
3-(Methylthio)propyl acetate
6-Epikarpoxanthin
MG(15:0/0:0/0:0)
(2E)-21-hydroxyhenicos-2-enoic acid
Squamone
Perulactone
Baliospermin
Dihydro-5-(2-octenyl)-2(3H)-furanone
Fistulosin
12'-apo-β-carotenal
LPA(0:0/16:0)
Parsonsine
Capsorubin
N-(4-{[(2-Amino-4-oxo-1,4,5,6,7,8-hexahydro-6-pteridinyl)methyl]amino}benzoyl)-γ-glutamyl-γ-glutamylglutamic acid
LSM-36486
Momordol
DG(15:0/16:1(9Z)/0:0)
Ala-Leu-Asp-Gly
5-Heptadecyl-1,3-benzenediol
Jasmonic acid
PE(18:2(9Z,12Z)/16:0)
4-Hydroxy-6-methyl-3-(1-oxobutyl)-2H-pyran-2-one
2,2,6,7-Tetramethylbicyclo[4.3.0]nona-1(9),4-dien-8-one
2-(4-Allyl-2,6-dimethoxyphenoxy)-1-(3,4-methylenedioxyphenyl)-1-propanol
Goyaglycoside C
Yamogenintetroside B
7-hydroxy-5-methoxy-6,8-dimethylisoflavone
N-cis-Feruloyltyramine
4-methyl-N-[2-[6-methyl-3-(methylthio)-1,2,4-triazin-5-yl]ethenyl]aniline
Hexadecasphinganine
2,6-Cyclolycopene-1,5-diol
Coronaric acid
Cryptocaryol E
Lycopersiconol
LSM-5734
Isolinderanolide
3-Hydroxyolean-12-en-28-oate
Glycol palmitate
Dihydrocapsaicin
(11S)-11-hydroperoxylinoleic acid
Pratenol A
Kaempferol 3-O-α-L-rhamnofuranoside
ω-hydroxy-15-methylpalmitic acid
Podecdysone B
Quinine
2-[4-(3,4-dihydro-1H-isoquinolin-2-yl)-4-oxobutyl]benzo[de]isoquinoline-1,3-dione
Gypsogenin 3-O-b-D-glucuronide
Neoflavan
Clerosterol 3-glucoside
Convallagenin A
(3S)-3-hydroxy-L-ornithine
Hypercalin B
(S)-chaulmoogric acid
Ethyl 1-[2-[3-(N-ethylanilino)propylamino]-3,4-dioxocyclobuten-1-yl]piperidine-4-carboxylate
Glycol stearate
Kaempferol 3-xylosylglucoside
4-[5-(4-ethoxyphenyl)-3-isoxazolyl]-N-(3-methoxyphenyl)butanamide
Tyrosyl-Proline
LSM-33458
Gniditrin
Capsaicin
6'',7''-dihydro-5',5'''-dicapsaicin
Castasterone 23-O-α-D-glucoside
2-Acetamido-2-deoxy-3-O-β-D-galactopyranosyl-1-O-{hydroxy[(hydroxy{[(2Z,6Z,10Z,14Z,18Z,22Z,26Z,30Z,34E,38E)-3,7,11,15,19,23,27,31,35,39,43-undecamethyl-2,6,10,14,18,22,26,30,34,38,42-tetratetracontaundecaen-1-yl]oxy}phosphoryl)oxy]phosphoryl}-α-D-glucopyranose
N-[(2S,3S,6R)-6-[2-(4-cyclohexyltriazol-1-yl)ethyl]-2-(hydroxymethyl)oxan-3-yl]-2-morpholin-4-ylacetamide
(18R)-18-hydroxynonadecanoic acid
Pollinastanol
1-O-[(E)-hexadecen-1-yl]-sn-glycerol
Ent-16b,19-Kauranediol 19-acetate
3b-Pregnadienolone 3-[rhamnosyl-(1->4)-rhamnosyl-(1->4)-rhamnosyl-(1->4)-glucoside]
22-hydroxydocosanoate
Soyacerebroside I
Theasapogenol E
3-{[(2,3-Dihydroxypropoxy)(hydroxy)phosphoryl]oxy}-2-(palmitoyloxy)propyl (9E,12E)-9,12-octadecadienoate
Lyciumoside II
1,2-Di-(9Z,12Z,15Z-octadecatrienoyl)-3-(Galactosyl-α-1-6-Galactosyl-β-1)-glycerol
11-Hydroxyyangonin
3-(Hexopyranosyloxy)-2-(palmitoyloxy)propyl (9E,12E,15E)-9,12,15-octadecatrienoate
1-hexadecanoyl-2-octadecanoyl-sn-glycero-3-phospho-D-myo-inositol
Soyacerebroside I
(2S)-3-(β-D-Galactopyranosyloxy)-2-[(9Z,12Z)-9,12-octadecadienoyloxy]propyl (9Z,12Z,15Z)-9,12,15-octadecatrienoate
(2S)-3-{[6-O-(β-D-Galactopyranosyl)-β-D-galactopyranosyl]oxy}-2-(palmitoyloxy)propyl (9Z,12Z,15Z)-9,12,15-octadecatrienoate
Gingerglycolipid A
1,1'-[1,13-Tridecanediylbis(oxy)]bisbenzene
387122
C20 sphinganine
Hexacosanedioic acid
(2S)-3-{[6-O-(β-D-Galactopyranosyl)-β-D-galactopyranosyl]oxy}-1,2-propanediyl (9Z,12Z,15Z,9'Z,12'Z,15'Z)bis(-9,12,15-octadecatrienoate)
(-)-(6Z,12E,2S,3S,4R,5R,9S,11S,15R)-3-cinnamoyloxylathyra-6,12-diene-5,15-diol-14-one
N-(icosanoyl)ethanolamine
Lablaboside A
LysoPC(15:0)
Aspartyl-Phenylalanine
Nordihydrocapsaicin
Sphinganine 1-phosphate
1,2-di-(9Z,12Z-octadecadienoyl)-3-(β-D-galactosyl)-sn-glycerol
N-Phenylacetylphenylalanine
Tricoumaroyl spermidine
Cucurbitacin E
Coumaric acid
1-stearoyl-2-(8Z,11Z,14Z-icosatrienoyl)-sn-glycero-3-phosphoserine
Tuberoside
3-(Hexopyranosyloxy)-2-(palmitoyloxy)propyl (9E,12E,15E)-9,12,15-octadecatrienoate
Demethylphosphinothricin
Soyasaponin aa
PC(18:3(9Z,12Z,15Z)/18:3(6Z,9Z,12Z))
2-(3,4-dimethoxyphenyl)-1-(2,4,6-trihydroxyphenyl)ethanone
1-O-palmityl-2-desoxy-2-amino-sn-glycero-3-phosphate
2-O-palmitoyl-α,α-trehalose
Carnosifloside III
(3β,5α,11α,12β,14β)-11-Acetoxy-3-{[6-deoxy-3-O-methyl-β-D-allopyranosyl-(1->4)-2,6-dideoxy-3-O-methyl-β-D-arabino-hexopyranosyl-(1->4)-2,6-dideoxy-3-O-methyl-β-D-ribo-hexopyranosyl]oxy}-14-hydroxy-20-oxopregnan-12-yl (2E)-3-phenylacrylate
Cysteamine S-phosphate
(1''S,2''R,3a'S,3a''S,5'R,5''S,5'''S,6a'S,6a''R,9a''S,9b''S,14a''S,17a''S,19''R,19a''R,19b''S)-4',5'',5''',19'',19b''-Pentahydroxy-1'',3',4',5,5,5''',9a'',17a'',19a''-nonamethyl-3''',3a',3a'',4,4',4''',4b'',5,5'',5''',6',6'',6''',6a',6a'',7'',9'',9a'',9b'',10'',12a'',13'',14'',14a'',15'',17'',17a'',17b'',18'',19'',19a'',19b''-dotriacontahydro-3H,3'H-trispiro[furan-2,2'-cyclopenta[b]furan-5',12''-furo[3'',2'':3',4']cyclopenta[1',2':5,6]napht
Pheophytin a
Avocadene
Quinic acid
Palmitic Acid
Prenyl arabinosyl-(1->6)-glucoside
Mucronine A
6-{[(4R,5R)-4-(hydroxymethyl)thymin-5-yl]methyl}thymine
Homocapsaicin
2-O-α-L-rhamnosyl-α-L-rhamnosyl-3-hydroxydecanoyl-3-hydroxydecanoic acid
4-Hydroxy-3-methoxybenzenemethanol
Homodihydrocapsaicin
Quercitrin
Adenin-9-yl riburonosate
2,2,6,7-Tetramethylbicyclo[4.3.0]nona-1(9),4-dien-8-one
α-L-Fucp-(1→3)-[α-D-Manp-(1→6)-[β-D-Xylp-(1→2)]-β-D-Manp-(1→4)-β-D-GlcpNAc-(1→4)]-D-GlcpNAc
N-oleoyl-L-leucine
Glyceraldehyde
F1-CQ
F1-QC
CO
QU
CW
ZU
CM
ST

Supplementary Material

1 Supplementary Figures

Supplementary Figure 1. Heatmap of 158 pre-annotated compounds in *C. annuum* L. fruits during development.

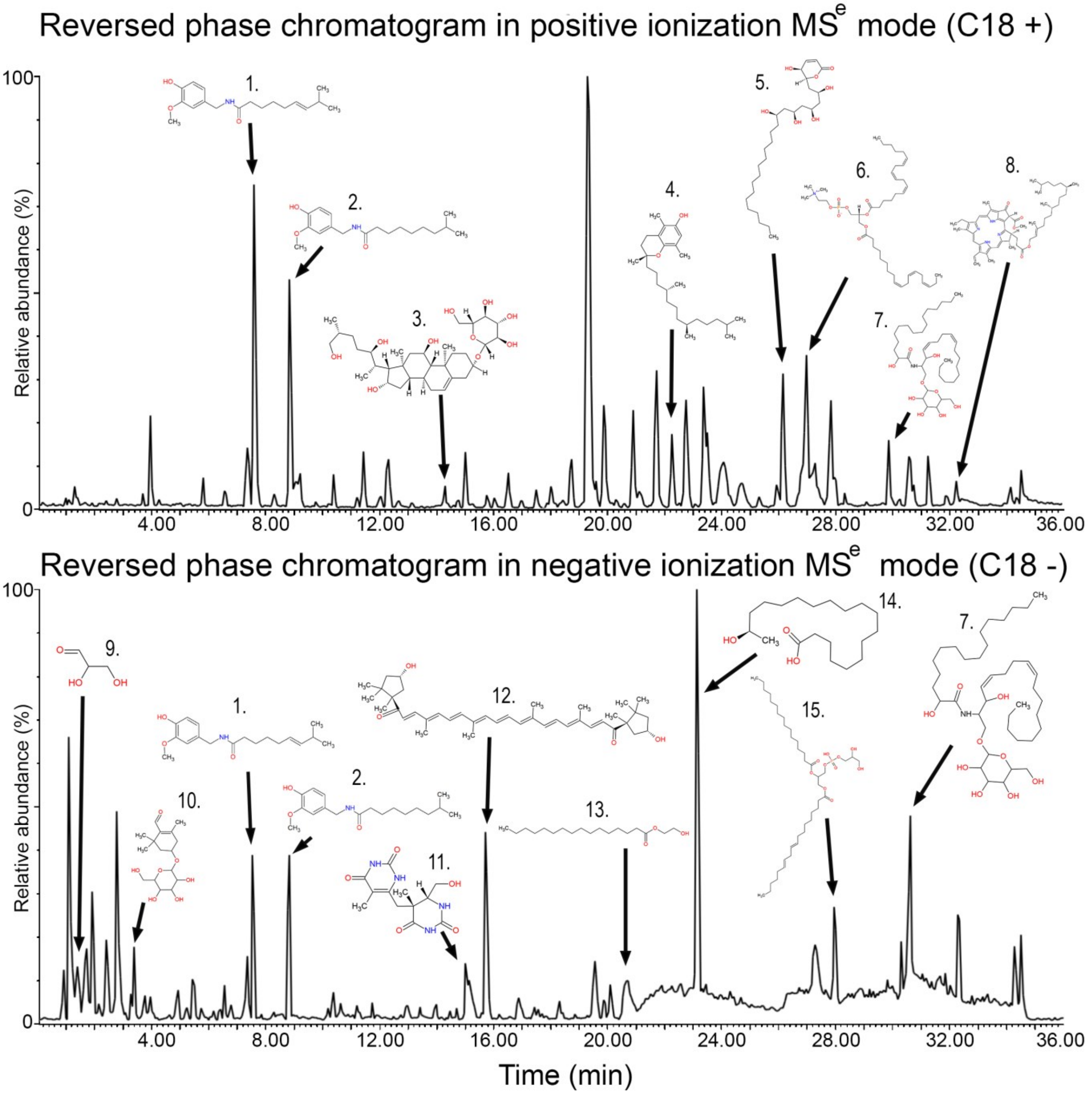

Supplementary Figure 2. Chromatogram of QC samples in positive and negative ionization mode. 1. Capsaicin; 2. Dihydrocapsaicin; 3. Heloside B; 4. β-tocopherol; 5. Cryptocaryol E; 6. PC(18:3(9Z,12Z,15Z)/18:3(6Z,9Z,12Z)); 7. Soyacerebroside I; 8. Pheophytin a; 9. Glyceraldehyde; 10. Aspartyl-Phenylalanine; 11. 6-{[(4R,5R)-4-(hydroxymethyl)thymin-5-yl]methyl}thymine; 12. Capsorubin; 13. Glycol palmitate; 14. (18R)-18-hydroxynonadecanoic acid; 15. 3-{[(2,3-Dihydroxypropoxy)(hydroxy)phosphoryl]oxy}-2-(palmitoyloxy)propyl (9E,12E)-9,12-octadecadienoate.
